# Under-canopy afforestation after 10 years: assessing the potential of converting monoculture plantations into mixed stands

**DOI:** 10.3389/fpls.2024.1340058

**Published:** 2024-03-14

**Authors:** Yuan Gao, Zhidong Zhang, Deliang Lu, Ying Zhou, Qiang Liu

**Affiliations:** ^1^ Hebei Agricultural University, College of Forestry, Baoding, China; ^2^ Qingyuan Forest CERN, National Observation and Research Station, Shenyang, Liaoning, China; ^3^ CAS Key Laboratory of Forest Ecology and Management, Institute of Applied Ecology, Shenyang, China

**Keywords:** *Pinus sylvestris* var. *Mongolica*, under-canopy afforestation, variable retention harvesting, growth and biomass allocation, leaf characteristics

## Abstract

Under-canopy afforestation using different tree species is a key approach in close-to-nature management to improve the structural and functional stability of plantation forests. However, current research on understory afforestation mainly focuses on the seedling stage, with limited attention to saplings or young trees. In this study, we evaluated the growth characteristics and leaf traits of 14-year-old *Pinus sylvestris* var. *Mongolica* trees under four different upper forest density (UFD) treatments: 0 trees/hm^2^ (canopy openness 100%, CK), 150 trees/hm^2^ (canopy openness 51.9%, T1), 225 trees/hm^2^ (canopy openness 43.2%, T2), and 300 trees/hm^2^ (canopy openness 28.4%, T3). We found that the survival rate of *P. sylvestris* in the T3 was significantly lower than in the other treatments, with a decrease of 30.2%, 18.3%, and 19.5% compared to CK, T1, and T2, respectively. The growth of *P. sylvestris* in the T1 treatment exhibited superior performance. Specifically, T1 showed a significant increase of 18.8%, 5.5%, and 24.1% in tree height, diameter at breast height, and crown width, respectively, compared to the CK. The mean trunk biomass ratio in the understory was significantly higher than that in full light by 15.4%, whereas the mean leaf biomass ratio was significantly lower by 12.3%. Understory *P. sylvestris* trees tended to allocate more biomass to the trunk at the expense of decreasing leaf biomass, which would facilitate height growth to escape the shading environment, although the promotion was relatively limited. Leaf length, leaf width, leaf area, leaf thickness, mesophyll tissue thickness, epidermis thickness, and leaf carbon content were the highest in the CK and tended to decrease with increasing UFD, indicating that a high-light environment favored leaf growth and enhanced carbon accumulation. In summary, young *P. sylvestris* trees adapted to moderate shading conditions created by the upper canopy, and the T1 treatment was optimal for the growth of understory *P. sylvestris*. This study provides insights into different adaptive strategies of young *P. sylvestris* trees to changes in light environment, providing practical evidence for under-canopy afforestation using light-demanding trees during pure plantation transformation.

## 1 Introduction

Chinese plantation area (80 million hm^2^) ranks first in the world, with pure plantations accounting for 85% (Chen et al., 2014). However, the simple forest structure of these plantations leads to potential problems such as low biodiversity, unstable ecosystems, and weak resilience. Furthermore, studies have shown that pure forests are more susceptible to large-scale pest and disease disasters, resulting in significant losses than mixed forests (Jactel et al., 2017; MacLean and Clark, 2021). Therefore, there is an urgent need to improve the stability and ecological functions of planted forests.

Close-to-nature management is a cultivation strategy that aims to convert pure plantation forests into near-natural forest ecosystems through various management measures, including the introduction of tree species, structural adjustment, promotion of natural regeneration, and protection of understory plant diversity (O’Hara, 2016; Schütz et al., 2016). These approaches have been shown to improve species diversity (Stokes et al., 2021), stand structure (Fang et al., 2021; Feng et al., 2022), carbon storage (Deng et al., 2023), and community succession (Chai and Tan, 2020). Under-canopy afforestation, which combines thinning and understory replanting of seedlings or saplings, is an effective method for transforming pure plantations into uneven-aged mixed forests. Compared to natural regeneration, under-canopy afforestation significantly shortens the time required for the conversion process (Paquette et al., 2006). Replanting seedlings under different light environments plays a crucial role in promoting forest succession. Therefore, it is necessary to study the growth and development of replanting seedlings under different light environments resulting from management interventions.

Light environment is an important factor in the growth and development of understory plants. The response to different light intensities can be observed at the plant level in individual development and biomass allocation. Generally, moderate light intensity favors the growth of woody plant seedlings, while excessive light intensity inhibits their growth. For example, Ma et al. (2015) found that seedlings of *Camptotheca acuminata* grown at 75% irradiance had significantly higher total biomass, height, and diameter than those grown under 100%, 25%, and 50% irradiance. In addition to height and diameter, biomass allocation reflects a plant’s strategy for efficiently acquiring resources and improving its competitive ability in response to its environment (Mediavilla and Escudero, 2010; Chmura et al., 2017). Some plants preferentially allocate biomass to stems under low-light conditions, such as in the understory or small forest gaps, to promote plant growth and escape from shaded environments (Van Hees and Clerkx, 2003; Poorter et al., 2012). Others allocate biomass to leaves to increase their ability to absorb and utilize photosynthetically active radiation (Nishimura et al., 2010; Boonman et al., 2020). On the one hand, the relative growth rates of different plant tissues and organs vary at different growth stages, resulting in alterations in the pattern of photosynthetic product allocation (Van De Vijver et al., 1993). On the other hand, the allocation of plant photosynthetic products is influenced by external environmental factors only when the nutrients obtained by the various plant organs are in a state of equilibrium (McConnaughay and Coleman, 1999). Evidently, the pattern of biomass allocation by plants under light-limiting conditions is not uniform, and the strategies of individual development and biomass allocation of different species in response to different light environments still need to be explored.

Leaves are the most sensitive organs of plants to changes in the external environment, and their external morphology, anatomical structure, and physiological characteristics can reflect the species’ response to environmental changes and resource competition (Poorter and Bongers, 2006). The adaptation of plant leaves to different light environments is diverse. Coble and Cavaleri (2015) found that leaf mass per area (LMA), leaf nitrogen content, and the carbon-to-nitrogen ratio (C/N) of *Acer saccharum* decreased significantly with decreasing light intensity, highlighting the significance of light availability on leaf morphology and chemistry. Yang et al. (2014) found that leaves from shaded sites had higher values for leaf size, specific leaf area, leaf nitrogen, and chlorophyll concentration per unit area than those from open sites, providing favorable conditions for species dominance in habitats with heterogeneous light conditions. However, most studies on plant adaptation to understory light environments have focused on containerized seedlings (Paquette et al., 2006; Liu et al., 2018; Fan et al., 2019), which may not fully reflect the adaptation of young trees at later stages.

In this study, we conducted an under-canopy afforestation experiment in 52-year-old *Larix principis-rupprechtii* plantations with different upper forest density (UFD) treatments. The growth and leaf characteristics of 14-year-old *P. sylvestris* trees were monitored under different UFD treatments. The specific aims were as follows: (1) to determine the feasibility of growth of *P. sylvestris* under different UFD treatments in the understory environment, and (2) to unveil the ecological adaptation mechanism of young *P. sylvestris* trees to the dynamic light conditions. We hypothesized that (1) young *P. sylvestris* trees would exhibit strong responses to the low-light environment resulting from shading in the upper stand. Specifically, we anticipated that the height growth and biomass allocation mechanisms of these trees would be closely linked, working together to enhance light capture and compete for light resources. (2) The UFD treatments would have varying effects on each response variable of young *P. sylvestris* trees in the understory, with the morphological structure of leaves being a significant factor influencing the growth and development of *P. sylvestris* in response to changes in light intensity. This study will offer insights into the adaptive responses of *P. sylvestris* young trees to variations in understory light conditions and provide scientific reference for the transition from pure plantation forests to uneven-aged mixed stands.

## 2 Materials and methods

### 2.1 Study site

The study was conducted in Saihanba Mechanized Forest Farm, located in Hebei Province, northern China (42°02′ - 42°36′N, 116°51′ - 117°39′E), at an altitude ranging from 1010.0 to 1939.9 m. The typical soils of the area are aeolian sandy soil, meadow soil, brown soil and gray forest soil. The climate is a typical temperate continental monsoon climate. It has an average annual temperature of -1.3°C, with extreme minimum and maximum temperatures of -43.3°C and 33.4°C, respectively. The area experiences an average snow cover for seven months, with an annual precipitation of 460 mm. The frost-free period lasts an average of 64 days. The total operating area of the forest farm is 94,000 hm^2^, with 73,000 hm^2^ designated as forest land, including 57,000 hm^2^ of planted forests and 16,000 hm^2^ of natural forests. The forest coverage rate is 80%, the total forest volume is 5.025 million m^3^, and the average annual growth rate is 9.7% (Xu et al., 2022). The main tree species include *L. principis-rupprechtii*, *Picea asperata*, *Betula platyphylla*, and *P. sylvestris* (Wu et al., 2023). The soil organic carbon content of the near-mature forests of *L. principis-rupprechtii* in this region is about 43.7 g/kg, total nitrogen 2.4 g/kg, total phosphorus 0.3 g/kg and total potassium 19.4 g/kg.

### 2.2 Experiment design

In 1970, an initial density of 5000 trees/hm^2^ of *L. principis-rupprechtii* was planted in the Saihanba Mechanized Forest Farm. To enhance the microenvironment of the forest and facilitate the establishment and growth of the replanted saplings, thinning was conducted on the upper layer of larch and retaining different tree densities. The retention densities for the upper layer of *L. principis-rupprechtii* consisted of four levels: control (clear-cut, providing a full-light environment without upper larch trees, CK), 150 trees/hm^2^ (Treatment 1, T1), 225 trees/hm^2^ (Treatment 2, T2), and 300 trees/hm^2^ (Treatment 3, T3).

In 2012, 4-year-old *P. sylvestris* saplings were planted under the *L. principis-rupprechtii* plantation (42 years old). The *P. sylvestris* saplings in the understory were spaced at 3 × 3 m, resulting in approximately 70-75 P*. sylvestris* per acre. All saplings were container-grown and planted using the “pit planting” method, ensuring consistent afforestation practices. In 2022, standard plots measuring 30 m × 20 m were established for each UFD treatment of *L. principis-rupprechtii*. The average height of the upper larch trees was 20.8 m, and the mean diameter at breast height (DBH) was 28.6 cm. Three replicates were set up for each treatment and control, resulting in 12 sample plots (i.e., 4 treatments × 3 replicates).

To demonstrate that there were differences in understory environments due to different UFD treatments between experimental gradients, canopy images were used to calculate canopy openness to better account for the effects of canopy structure on understory environments. Canopy images were captured using a wide-angle lens at a height of 2 m in different UFD treatments. The photographs were taken under overcast conditions to minimize glare from direct sunlight, following the method described by Beaudet and Messier (Beaudet and Messier, 2002). The canopy light environment data were obtained by analyzing the images using Hemiview software (Li et al., 2014). The results revealed canopy openness values of 51.9%, 43.2%, 28.4%, and 100% for T1, T2, T3, and CK, respectively.

### 2.3 Data collection

#### 2.3.1 Monitoring of young tree survival, growth, and biomass accumulation

At the end of the 2022 growing season, dead young trees within each standard plot were observed and recorded. The survival rate of young trees in each plot was estimated as the percentage of living individuals to the total number of individuals initially planted in each plot. Measurements were taken for all individual trees in each plot, including diameter at breast height (DBH, defined as 1.3 m above ground level in China), crown width, and tree height. Diameter tape, steel tape, and ultrasonic hypsometer (Vertex IV, Haglöf Sweden) were used for these measurements.

Based on the average DBH and height in each plot, one healthy tree was selected near each standard plot and then separated into different organs, including trunk, branch, leaf, and root, and their fresh weights were measured. Samples from each organ were collected and brought back to the laboratory, weighed, and dried in an oven at 85°C for at least 72 hours until a constant weight was achieved. The dry weight of each sample was measured, and the biomass of each tree organ was calculated based on the ratio of dry weight to fresh weight. The above-ground biomass was calculated as the sum of trunk, branch, and leaf biomass; the below-ground biomass represented the root biomass.

#### 2.3.2 Measurement of leaf morphology, nutrients, and anatomy

Following the principle of even sampling (Cornelissen et al., 2003), six individual *P. sylvestris* trees were selected from each plot to represent the average characteristics of the plot. These young trees were used for the determination of leaf indexes.

To account for the effects of light and other factors on branch and leaf growth in different orientations and crown positions, we collected current-year branches from the middle position of the crown on the south side of each selected tree. From each collected branch, leaf samples were randomly chosen. The fresh weight (FW) of the leaf samples was measured using an electronic balance with an accuracy of 0.001g. The leaf samples were then scanned using a leaf image analyzer (EP-leaf1000, China). Each leaf sample was scanned for the following characteristics: leaf length, leaf width, leaf length-to-width ratio, and leaf area. After the measurements were completed, each leaf sample was grouped together. The group was then dried in an oven at 85°C until a constant weight was achieved. The dried cluster was weighed to determine the leaf dry weight (LDW), leaf dry matter content (LDMC), and leaf mass per area (LMA) according to the method described by Cornelissen et al. (2003):


(1) $$LDMC_{i}=LDW_{i}/FW_{i}$$

(2) $$LMA_{i}=LDW_{i}/LA_{i}$$

In Equations (1, 2), i represents the ith leaf samples. LDMC means leaf dry matter content; LDW means leaf dry weight; FW means fresh weight; LMA means leaf mass per area; LA means leaf area.

The remaining leaves from the collected branches were extracted and stored in labeled envelopes. They were then dried in an oven at 85°C until a constant weight was achieved. The dried leaf samples were subsequently ground and crushed. The samples were sent to the laboratory for analysis after passing through a 0.3 mm (60 mesh) sieve. The content of carbon (C), nitrogen (N), and phosphorus (P) in the leaf was determined using methods described by Jones and Case (1990). The ratios of carbon to nitrogen (C/N), carbon to phosphorus (C/P), and nitrogen to phosphorus (N/P) in the leaf were expressed as mass ratios.

The current-year branches from the middle of the canopy on the south side of each tree were sampled. To avoid positional effects at the tip and base of the twigs, we only collected leaf samples from the middle part of the twigs (Zhao et al., 2008). Three fresh leaves of moderate thickness were carefully selected and immediately placed into FAA solution (38% formaldehyde, glacial acetic acid, 70% alcohol, 5:5:90, v/v/v). The leaves were left in the solution for at least 48 hours to ensure proper fixation. After fixation, the field-fixed leaves were sectioned using the paraffin sectioning method. The sections obtained were 8-10 μm thick and were double-stained with senna red and solid green. They were then sealed with neutral gum. Leaf thickness, mesophyll tissue thickness, and epidermis thickness were observed and measured using an upright microscope (OLYMPUS BX41) and an image analysis system (Toupview) after sectioning.

### 2.4 Data analysis

One-way ANOVA and Duncan’s multiple comparisons were employed to assess the differences in survival, growth, and biomass of young *P. sylvestris* trees, leaf morphology, nutrient content, and anatomical characteristics across different UFD treatments. Principal component analysis (PCA) was utilized to visualize the overall coordination and correlation between tree growth and leaf traits in the various canopy treatments. The experimental data were analyzed using ANOVA in SPSS 25.0 statistical software, followed by Duncan’s test (p< 0.05) for post-hoc comparisons. PCA and all figures were generated using R statistical software. The FactoMineR and factoextra packages were employed for the PCA analysis. All data are presented as mean ± standard error.

## 3 Results

### 3.1 Effects of UFD treatments on tree survival, growth and biomass allocation

**Figure 1:**
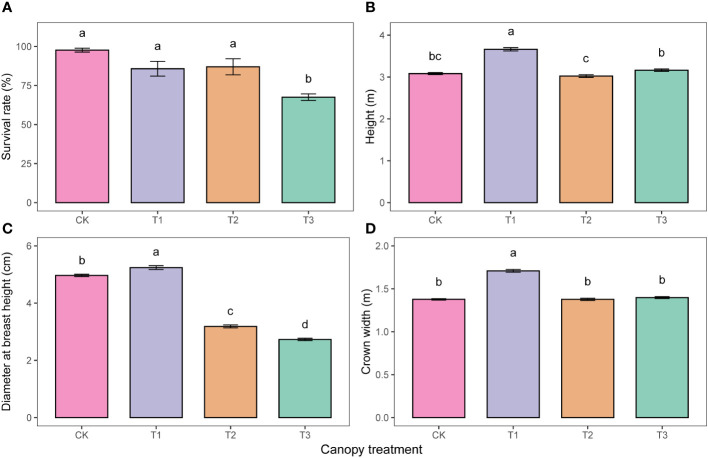
Survival **(A)** and growth **(B-D)** under different upper forest density (UFD) treatments. Different lowercase letters indicate significant differences among UFD treatments (P< 0.05). Each value is the mean ± stand error. CK: under full-light conditions; T1: UFD was 150 trees/hm^2^; T2: UFD was 225 trees/hm^2^; T3: UFD was 300 trees/hm^2^.

The results of the ANOVA indicated a significant effect (P<0.05) of the UFD treatments on the survival, growth, leaf morphology, leaf anatomy, and leaf nutrients of young *P. sylvestris* trees (**Table 1**). The survival rate of *P. sylvestris* in the T3 was only 67.5%, 30.2%, 18.3%, and 19.5% lower than the CK, T1, and T2, respectively (**Figure 1A**). As the UFD increased, the growth of under-canopy *P. sylvestris* initially increased and then decreased. The maximum values of tree height, DBH, and crown width were observed in the T1, significantly higher than in the other treatments (**Figures 1B–D**).

**Table 1:** The ANOVA results for the effects of upper forest density on tree growth, biomass, leaf morphology, anatomy, and nutrients.

Category	Measure variable	*df*	F-value	P-value
Growth	Survival rate	3	11.42	<0.01**
	Height	3	76.49	<0.01**
	DBH	3	666.72	<0.01**
	Crown width	3	163.82	<0.01**
Biomass	Total biomass	3	12.92	<0.01**
	Above-ground biomass	3	11.30	<0.01**
	Below-ground biomass	3	2.00	0.19
	Trunk biomass ratio	3	8.05	<0.01**
	Branch biomass ratio	3	1.33	0.33
	Leaf biomass ratio	3	4.39	<0.05*
	Root biomass ratio	3	0.43	0.74
	Above-ground biomass ratio	3	0.43	0.74
Leaf morphology	Leaf length	3	4.61	<0.01**
	Leaf width	3	13.73	<0.01**
	Leaf length-to-width ratio	3	0.90	0.44
	Leaf area	3	12.96	<0.01**
	LMA	3	5.58	<0.01**
	LDMC	3	13.10	<0.01**
Leaf anatomy	Leaf thickness	3	30.33	<0.01**
	Leaf tissue thickness	3	28.43	<0.01**
	Epidermis thickness	3	19.33	<0.01**
Leaf nutrients	Leaf N content	3	19.15	<0.01**
	Leaf P content	3	13.13	<0.01**
	Leaf C content	3	16.74	<0.01**
	Leaf N/P ratio	3	5.29	<0.01**
	Leaf C/N ratio	3	27.75	<0.01**
	Leaf C/P ratio	3	18.23	<0.01**

**: P<0.01; *: P<0.05.

**Figure 2:**
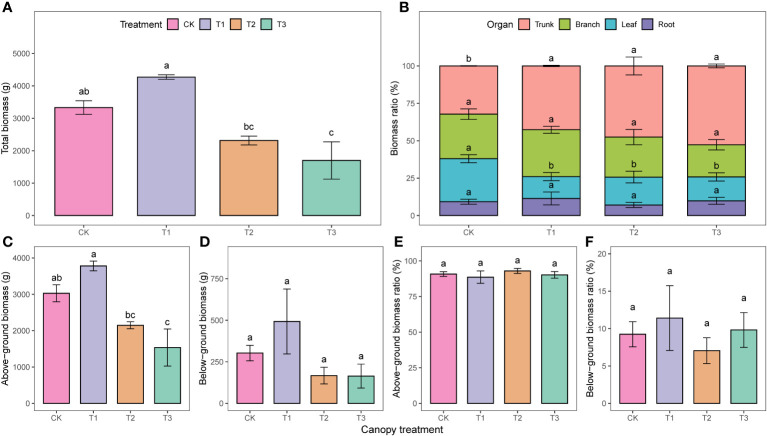
Biomass accumulation (**A, C, D**) and allocation (**B, E, F**) under different upper forest density (UFD) treatments. Different lowercase letters indicate the significant differences among UFD treatments (P< 0.05). Each value is the mean ± stand error. CK: under full-light conditions; T1: UFD was 150 trees/hm^2^; T2: UFD was 225 trees/hm^2^; T3: UFD was 300 trees/hm^2^.

The total and above-ground biomass followed a similar trend with different UFD treatments. There were no significant differences between the CK and T1, but both were significantly higher than the T2 and T3 (**Figures 2A, C**). The proportion of trunk biomass to total biomass in the CK was 10.4%, 15.3%, and 20.4% lower than T1, T2, and T3, respectively (**Figure 2B**). However, the leaf biomass ratio in the CK was significantly higher than in the other treatments. There were no significant differences in the above-ground and below-ground biomass ratios among the treatments (p>0.05, **Figures 2E, F**).

### 3.2 Effects of UFD treatments on leaf morphology, anatomy, and nutrients

**Figure 3:**
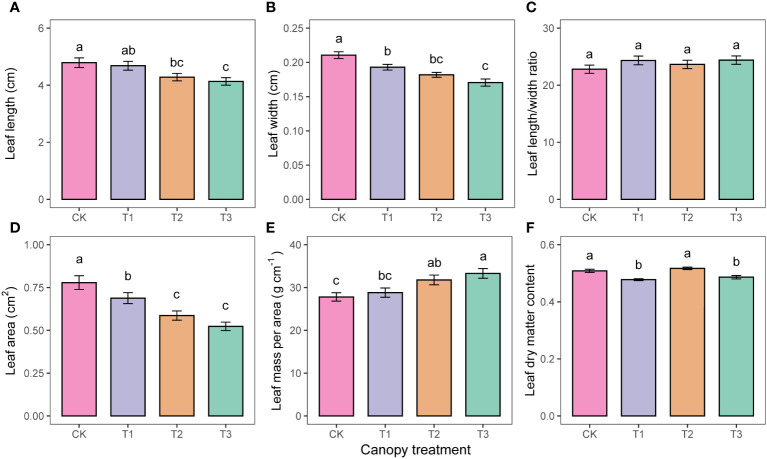
Differences in leaf morphology **(A–F)** under different upper forest density (UFD) treatments. Different letters indicate significant differences (P< 0.05) among treatments. Each value is the mean ± stand error. CK: under full-light conditions; T1: UFD was 150 trees/hm^2^; T2: UFD was 225 trees/hm^2^; T3: UFD was 300 trees/hm^2^.

Mean leaf length, leaf width, and leaf area all decreased with increasing UFD (**Figure 3A, B, D**). The leaf area of CK was 13.2%, 32.8%, and 48.9% higher than that of T1, T2, and T3, respectively. However, LMA showed the opposite trend, increasing with increasing UFD (**Figure 3E**). Leaf length-to-width ratio did not differ significantly among treatments (p>0.05, **Figure 3C**).

**Figure 4:**
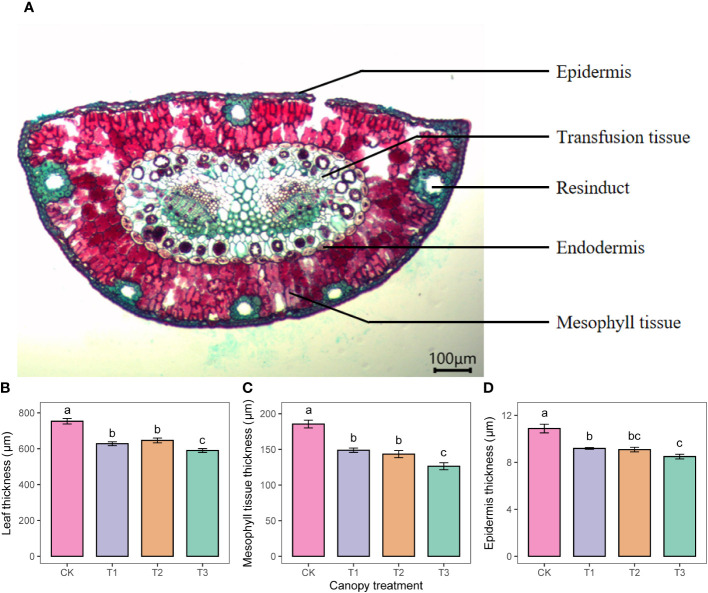
Anatomical structures of *P. sylvestris* leaves **(A)** and statistical analyses on the thickness of leaf tissue **(B-D)** under different upper forest density (UFD) treatments. Different letters indicate significant differences (P< 0.05) among treatments. Each value is the mean ± stand error. CK: under full-light conditions; T1: UFD was 150 trees/hm^2^; T2: UFD was 225 trees/hm^2^; T3: UFD was 300 trees/hm^2^.

Anatomical profiles showed that *P. sylvestris* leaves were semicircular in cross-section, with undifferentiated mesophyll tissue consisting of irregularly arranged but tightly packed cells. Multiple resin ducts were between the leaf tissue cells (**Figure 4A**). As the UFD increased, the thickness of the leaf, mesophyll tissue, and epidermis showed an overall decreasing trend (**Figures 4B–D**).

**Figure 5:**
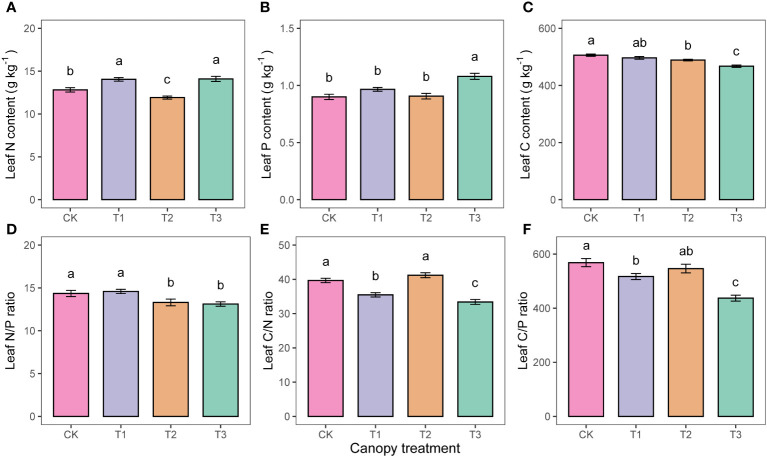
Differences in leaf nutrients **(A–F)** under different upper forest density (UFD) treatments. Different letters indicate significant differences (P< 0.05) among treatments. Each value is the mean ± stand error. CK: under full-light conditions; T1: UFD was 150 trees/hm^2^; T2: UFD was 225 trees/hm^2^; T3: UFD was 300 trees/hm^2^.

The pattern of variation for leaf nutrient indicators with UFD treatments was inconsistent. Leaf C content decreased with increasing UFD (**Figure 5C**), whereas leaf P content of the T3 was significantly higher than that of the other treatments (**Figure 5B**). Leaf nutrient stoichiometric ratios indicated that N/P was not significantly different between CK and T1, but both were significantly higher than T2 and T3 (**Figure 5D**). The C/N was significantly higher in CK, T1, and T2 than in T3 (**Figure 5E**).

### 3.3 Relationships among young tree characteristics in different UFD treatments

**Figure 6:**
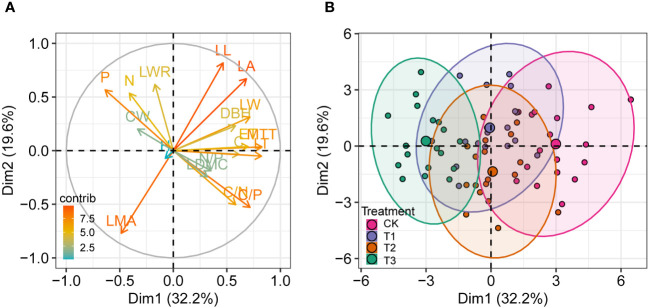
Principal component analysis (PCA) of different variables of *P. sylvestris* under different upper forest density (UFD) treatments. Tree and leaf trait values are loaded on the first two PC axes. **(A)** Contributions of variables to PCs. **(B)** Grouping of samples with different treatments. The “contrib” value represents the contribution to the principal components. H, height; DBH, diameter at breast height; CW, crown width; LL, leaf length; LW, leaf width; LWR, LL/LW ratio; LA, leaf area; LMA, leaf mass per area; LDMC, leaf dry matter content; N, leaf N content; P, leaf P content; C, leaf C content; N/P, leaf N/P ratio; C/N, leaf C/N ratio; C/P, leaf C/P ratio; LT, leaf thickness; MTT, mesophyll tissue thickness; ET, epidermis thickness; CK, under full-light condition; T1, UFD was 150 trees/hm^2^; T2, UFD was 225 trees/hm^2^; T3, UFD was 300 trees/hm^2^.

The principal component analysis based on 18 variables of *P. sylvestris* under different UFD treatments showed that the first two principal components explained 51.8% of the total phenotypic variance (**Figure 6**). The first dimension (Dim 1) explained 32.2% of the total variance. Leaf thickness, mesophyll tissue thickness, leaf width, and leaf area had significant positive correlations with the first dimension, all of which are leaf morphology and anatomy indicators, suggesting that the first principal component mainly reflects the variation of leaf structure. The second dimension (Dim 2) explained 19.6% of the total variance. LMA, leaf length-to-width ratio, and leaf C/N ratio had significant correlations with the second dimension, all of which are leaf functional traits (**Figure 6A**). The *P. sylvestris* samples of the CK differed significantly from those of the T3, of which CK had positive coordinates, and T3 had negative coordinates on the first dimension (**Figure 6B**). However, there was no significant separation from the T1 and T2, and there was a degree of clustering.

## 4 Discussion

### 4.1 Response of growth characteristics to different UFD treatments

*P. sylvestris* young trees exhibited the highest survival rate under full light conditions. However, under the T3, characterized by extreme low light conditions, there was a significant decrease in the survival of *P. sylvestris*. This finding aligns with the results of Zhang et al. (2013) on *Pinus koraiensis* and *Quercus mongolica*, where reduced light intensity resulted in decreased survival of light-demanding species. However, the growth of *P. sylvestris* young trees in the understory did not follow the same pattern as survival. In our study, tree height, DBH, and crown width were significantly higher in the T1 compared to the other treatments. This suggests that the mild shading resulting from a lower UFD was conducive to promoting the growth of *P. sylvestris* young trees in the understory. This phenomenon, known as the shade-avoidance response (Fiorucci and Fankhauser, 2017), indicates that the species adapts to a low-light environment by altering its morphology. However, when the UFD exceeded 150 plants/hm^2^, the growth of young trees in the understory began to exhibit a decreasing trend. It has been confirmed that weak light resulted in low photosynthesis, reducing the plant’s carbon assimilation potential (Sharkey et al., 2007; Dai et al., 2009). Consequently, the photosynthetic carbon assimilation products could not meet the plant’s normal growth requirements, leading to growth suppression. This finding is consistent with previous studies on *Torreya grandis* seedlings (Tang et al., 2015).

The total biomass was significantly higher in the CK and T1 (strong light environment) compared to T2 and T3 (weak light environment). This finding confirms previous studies showing a positive correlation between strong light conditions and dry matter accumulation (Toledo-Aceves and Swaine, 2008; Lu et al., 2018). The proportion of trunk biomass to total biomass gradually increased with increasing UFD. This suggests that *P. sylvestris* young trees preferentially allocate biomass to the trunk to enhance tree height growth and improve their ability to compete for light resources in the presence of shading from the upper canopy. This finding aligns with our initial hypothesis and supports the acclimation strategy, which suggests that plants preferentially allocate biomass to organs that most efficiently acquire light, water, and nutrients. It also suggests that biomass allocation within a plant may involve an optimization process in response to stress (Garnier, 1991). However, there were no significant differences in above- and below-ground biomass ratios among the UFD treatments. This indicates that the response of biomass allocation in young *P. sylvestris* trees to the light environment is limited. Additionally, biomass allocation is not fixed and varies with time, environment, and species (Poorter et al., 2012). Therefore, long-term monitoring may be necessary to further understand the treatment effects.

Considering the observed differences in morphology among young *P. sylvestris* trees under different light conditions, it is evident that the variation in biomass allocation is relatively smaller than in plant morphology, but tree morphology exhibits greater plasticity than biomass allocation in response to the light environment. This finding is consistent with previous studies on species such as *Fagus sylvatica* seedlings (Curt et al., 2005) and *Camellia oleifera* (Zhang et al., 2022b).

### 4.2 Response of leaf characteristics to different UFD treatments

In this study, mean leaf length, leaf width, and leaf area decreased, but LMA increased with increasing UFD. However, previous studies on *Acer saccharum* (Coble and Cavaleri, 2015), *Camellia oleifera* (Zhang et al., 2022b), and *Medicago sativa* (Tang et al., 2022) found that plants typically increase leaf area to capture more light resources by expanding the light-receiving surface, decreasing LMA in shaded environments. This may be a difference caused by different life types of trees. Structural diversification in response to different light availability was smaller in leaves of evergreen conifers, as noted in previous studies on conifers (Youngblood and Ferguson, 2003; Wyka et al., 2007, 2012) and other evergreen species (Valladares et al., 2000). In contrast, deciduous broadleaf species, with their shorter leaf lifespan, typically invest more dry matter into plant growth and leaf area expansion. Therefore, inferring the light-trapping capacity of conifers based solely on their morphological size is limited. Furthermore, there were no significant differences in the leaf length and width ratio among the different upper stand density treatments. This indicates that the leaves of *P. sylvestris* young trees adapt to low-light environments by altering their size rather than their shape.

Previous studies on leaf structure have mainly focused on broadleaf species, revealing that plants tend to reduce leaf thickness, particularly the thickness of palisade tissues, in response to decreased light intensity as an adaptation to low-light environments (Kong et al., 2016; Zhang et al., 2022a, b). In our study, which focused on a coniferous species, the mesophyll tissue did not differentiate into palisade and spongy tissue. However, our findings also demonstrated that *P. sylvestris*, similar to other broadleaf species, exhibited a gradual decrease in leaf thickness, mesophyll tissue thickness, and epidermis thickness with decreasing light intensity. The thickening of the epidermis under high light intensity may reduce stomatal conductance, enhancing water use efficiency. These changes can protect photosynthetic tissues under conditions of high light intensity and drought (Hanba et al., 2002; Ivancich et al., 2012). Conversely, leaf thickness and mesophyll tissue thickness are reduced under low light conditions. Previous studies have indicated that thinner leaves have a higher likelihood of light interception, but this structural adaptation can be detrimental to CO_2_ fixation and transport (Terashima et al., 2011).

The average leaf C content increased with increasing light intensity, which supports the findings of Coble and Cavaleri (2015) that higher light levels lead to increased carbon investment in leaf structures during adaptation. The leaf N/P ratio can indicate the nutrient limitation status of plants in their environment (Güsewell, 2004). Generally, plant growth is limited by P when the N/P ratio is greater than 16, N when it is less than 14, and both elements when it falls in between (Koerselman and Meuleman, 1996). In our study, under the CK and T1 treatments, which experienced higher light intensity, the N/P ratio of young *P. sylvestris* leaves was significantly higher than that of *P. sylvestris* under low light conditions. On the other hand, the average N/P ratio of the four treatments was about 14. It indicated that the growth of *P. sylvestris* was more limited by N in our study environment. Further investigation reveals that the variation in N and P contents under the canopy is influenced by both the plant’s inherent supply-demand dynamics and the ability of the mixed forest to modify soil conditions (Wang et al., 2013; Chen et al., 2022). A previous study reported a total soil nitrogen content of 0.60 ± 0.02 g/kg in this area (Niu et al., 2019), indicating a nutrient-poor state according to the nutrient classification standard of the second national soil census. Therefore, the observed nitrogen limitation in this region may be attributed to the depletion of nitrogen nutrients in the soil. In this study, although the C/N of CK, T1, and T2 under better light conditions was significantly higher than that of T3 under low light conditions, the values of C/N did not have a significant trend with decreasing light intensity. Furthermore, it has been demonstrated that the variation in leaf C/N was primarily influenced by soil water content (Lu et al., 2023). Therefore, the changes in leaf nutrient and stoichiometric ratios may be more closely associated with soil physicochemical properties.

### 4.3 Relationships among various characteristics in different UFD treatments

Different UFD treatments were distributed in different regions of the PCA score plot, with significant differences observed between CK and T3 along the first principal component axis. Leaf anatomy and morphology exhibited higher loading coefficients in the first principal component. This finding emphasizes the importance of leaf structure, which includes both external morphological features and internal anatomical structures, as a key indicator for the adaptation of young *P. sylvestris* trees to diverse understory environments and resource utilization. This observation aligns with our initial hypothesis. However, T1 and T2 showed considerable overlap in the PCA score plot. This finding suggests that understory *P. sylvestris* young trees are well adapted to moderate shade environments without much variability.

Previous studies on *Tetrastigma hemsleyanum* (Dai et al., 2009) have found that photosynthetic activity and growth are depressed under light intensities greater than 50% shade. Carbon assimilation is limited and plant growth decreases under irradiance levels lower than 75% shade. As can be seen, optimal light intensity varies from species to species. In this study, when UFD is less than 225 trees/hm^2^, the inhibitory effect of upper canopy shading on the growth and development of understory *P. sylvestris* young trees decreases. Therefore, it is not advisable to maintain a high UFD when planting *P. sylvestris* young trees under the forest canopy. Furthermore, the light requirements of plants vary at different developmental stages. Thus, conducting long-term monitoring of the growth status of understory seedlings is necessary to optimize forest stand structure. These results provide a reference for regulating interspecific relationships and promoting the growth and development of *P. sylvestris* young trees in mixed forests under the forest canopy.<5. Conclusion and management implication.

This experiment investigated the effects of UFD treatments on the individual development, biomass allocation, leaf morphology, nutrients, and anatomical structure of young *P. sylvestris* trees. We found that *P. sylvestris* young trees exhibited better growth under the T1, with higher tree height, DBH, crown width, and total biomass, which supported that it would be practicable for under-canopy afforestation using *P. sylvestris* as long as sufficient light transmittance could be ensured. Under shading conditions, *P. sylvestris* young trees allocated more photosynthetic products to the main trunk area, improving their light competition and survival ability. Light availability had greater impacts on tree morphology than biomass allocation. However, long-term monitoring is necessary during the young tree stage to identify potential trends in response changes. The introduction of *P. sylvestris* into pure larch forests can optimize stand structure, including species composition and vertical layering, and promote near-natural succession in these monoculture forests.

## Data availability statement

The original contributions presented in the study are included in the article/supplementary material. Further inquiries can be directed to the corresponding authors.

## Author contributions

YG: Data curation, Formal Analysis, Investigation, Writing – original draft, Writing – review & editing. QL: Funding acquisition, Writing – review & editing, Methodology, Supervision. DL: Funding acquisition, Methodology, Supervision, Writing – review & editing. YZ: Formal Analysis, Investigation, Writing – review & editing. ZZ: Funding acquisition, Writing – review & editing, Project administration.
